# Supplementary material for: Benzene and NO2 Exposure during Pregnancy and Preterm Birth in Two Philadelphia Hospitals, 2013–2017
Source: Int J Environ Res Public Health. 2022 Aug 19;19(16):10365. doi: 10.3390/ijerph191610365 (PMC9408580; doi:10.3390/ijerph191610365)
Supplement: Supplementary file 1 [file ijerph-19-10365-s001.zip › ijerph-1841839-supplementary.pdf]

Table S1. Unadjusted and adjusted[a] odds ratio (OR) of a standard deviation increment increase in Benzene and trimester-level NO_2 exposures with preterm birth (PTB) outcomes including spontaneous (sPTB) and medically indicated (mPTB).

	**Birth outcome: PTB OR (95% CI)**	**Birth outcome: sPTB OR (95% CI)**	**Birth outcome: mPTB OR (95% CI)**
	First Trimester		
Unadjusted NO2	0.998 (0.95, 1.05)	1.01 (0.952, 1.08)	0.976 (0.903, 1.05)
Adjusted NO2	1.03 (0.98, 1.09)	1.03 (0.97, 1.11)	1.02 (0.95, 1.11)
Unadjusted Benzene	0.96 (0.91, 1.01)	1.00 (0.94, 1.06)	0.89 (0.82, 0.97)*
Adjusted Benzene	1.01 (0.96, 1.07)	1.04 (0.97, 1.12)	0.959 (0.88, 1.04)
	Second Trimester		
Unadjusted NO2	0.935 (0.890, 0.983)*	0.965 (0.906, 1.03)	0.896 (0.828, 0.969)*
Adjusted NO2	0.951 (0.902, 1.00)	0.960 (0.897, 1.03)	0.940 (0.866, 1.02)
Unadjusted Benzene	0.96 (0.91, 1.01)	1.00 (0.94, 1.06)	0.89 (0.82, 0.97)*
Adjusted Benzene	1.02 (0.964, 1.08)	1.05 (0.974, 1.13)	0.972 (0.893, 1.06)
	Third Trimester		
Unadjusted NO2	0.959 (0.907, 1.01)	0.972 (0.905, 1.04)	0.926 (0.850, 1.01)
Adjusted NO2	0.965 (0.910, 1.02)	0.950 (0.881, 1.02)	0.968 (0.884, 1.06)
Unadjusted Benzene	0.96 (0.91, 1.01)	1.00 (0.94, 1.06)	0.89 (0.82, 0.97)*
Adjusted Benzene	1.01 (0.951, 1.07)	1.04 (0.963, 1.13)	0.965 (0.882, 1.06)

[a]Adjusted models were logistic mixed models adjusting for age, race, BMI, nulliparity, insurance status, and census tract level percent below poverty. Hospital and census tracts were included as random intercepts. Adjusted models mutually adjusted for the other pollutant.

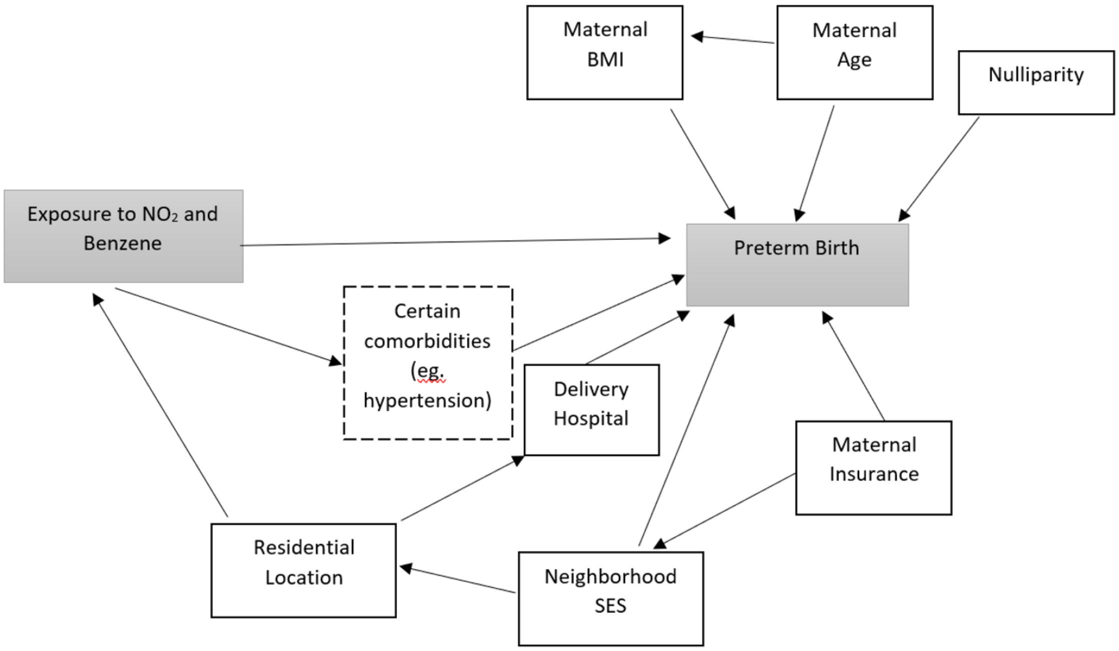

Figure S1. Directed acyclic graph demonstrating the assumed relationships between different socioeconomic and health factors that may have an effect when studying the relationship between NO_2 and benzene exposure to preterm births. Certain medical comorbidities may be on the causal pathway between air pollution exposure and preterm birth.
